# Arrhythmogenic left ventricular cardiomyopathy

**DOI:** 10.1259/bjrcr.20190079

**Published:** 2020-02-12

**Authors:** Seyedeh Mojdeh Mirmomen, Andrew Jay Bradley, Andrew Ernest Arai, Arlene Sirajuddin

**Affiliations:** 1 National Heart, Lung and Blood Institute, National Institutes of Health, Bethesda, MD, USA

## Abstract

Arrhythmogenic ventricular cardiomyopathy (AVC) is a heritable heart muscle disorder characterized by fibrofatty infiltration of the myocardium. Intramyocardial fat deposition is considered arrhythmogenic and predisposes patients to life-threatening arrhythmias and sudden cardiac death. The classic subtype of AVC is characterized by fibrofatty replacement of the right ventricular myocardium (*i.e.* arrhythmogenic right ventricular cardiomyopathy). In advanced cases of arrhythmogenic right ventricular cardiomyopathy, the left ventricle may be involved as well. Predominantly left ventricular involvement by AVC is exceedingly rare and lack of specific diagnostic criteria as well as its potential cardiotoxic effect make its diagnosis challenging and of high importance.

## Clinical presentation

A 59-year-old female presented with an abnormal preoperative electrocardiogram (ECG), suspicious for possible myocardial ischemia. She denied chest pain, dyspnea, orthopnea, palpitations and edema. She reported occasional dizziness. Her past medical history was remarkable for hypertension, hyperlipidemia, migraine headaches, and oophorectomy. She was also a current smoker (1/2 pack per day). She had a family history of coronary artery disease.

Because the ECG was suspicious for possible myocardial ischemia as well as her multiple risk factors for coronary artery disease, she underwent cardiac catherization which showed no coronary artery disease. However, the cardiac catherization showed that the left ventricular ejection fraction (LVEF) was severely decreased, visually estimated to be 35% on the ventriculogram. She began medical therapy and on follow-up echocardiogram her LVEF improved to 65%. She was followed with serial echocardiograms over the last 15 years, however the LVEF eventually decreased to approximately 35% again.

Her most recent transthoracic echocardiogram revealed a normal sized left ventricle with severely decreased systolic function with a LVEF of 30–35%, grade 1 diastolic dysfunction and global hypokinesis with regional variability. The right ventricle was normal in size and function. Aside from mild mitral regurgitation, the valves were normal. Pulmonary artery systolic pressure was normal. A recent stress test was negative for ischemia. Cardiac MRI was requested for further evaluation.

## Imaging findings

**Figure 1.**
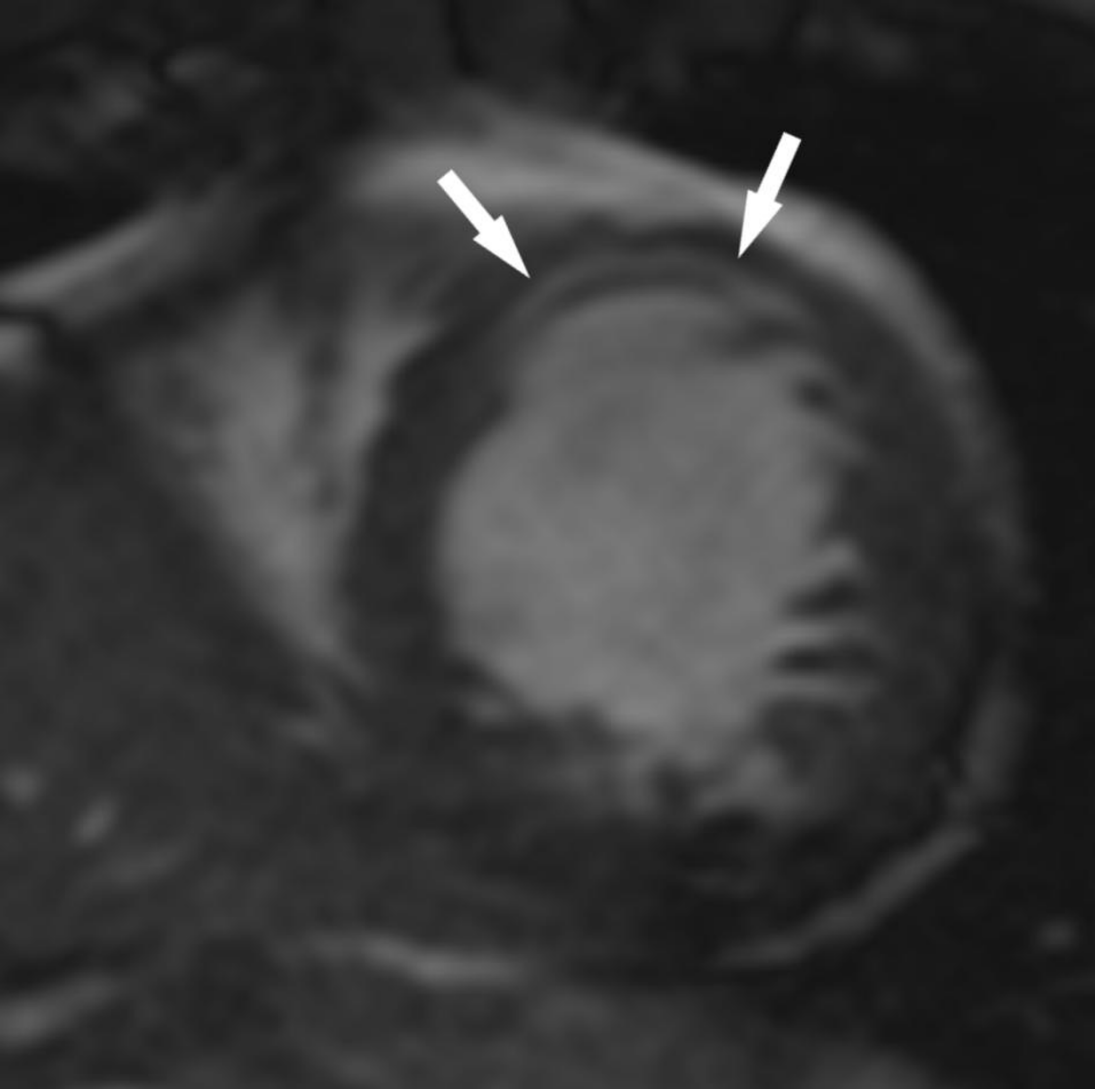
Short-axis steady state free precession (SSFP) cine image of the left ventricle shows one example of several midwall areas of hyperintensity within the mid septal and anterior walls (arrows).

The patient underwent cardiac MRI at 1.5 T (Siemens Healthineers, Erlangen, Germany) for further evaluation. Steady-state free precession cine images (Figure 1) demonstrated severely reduced global systolic function (LVEF 35%). There was global hypokinesis with superimposed akinesis of the anterior wall and septum. The akinetic anteroseptal and anterior segments had midwall hyperintensity which was suspicious for intramyocardial fat.

**Figure 2.**
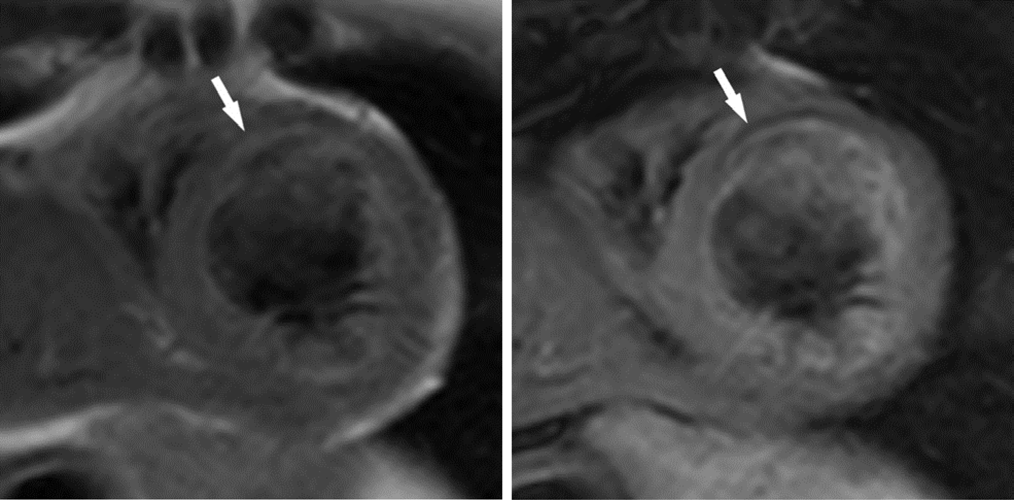
Short-axis *T*_2_ weighted turbo spin echo black blood images without (left) and with (right) fat suppression. Arrows indicate one example of an area of signal that nulls with the application of a fat suppression pulse which is compatible with intramyocardial fat.

**Figure 3.**
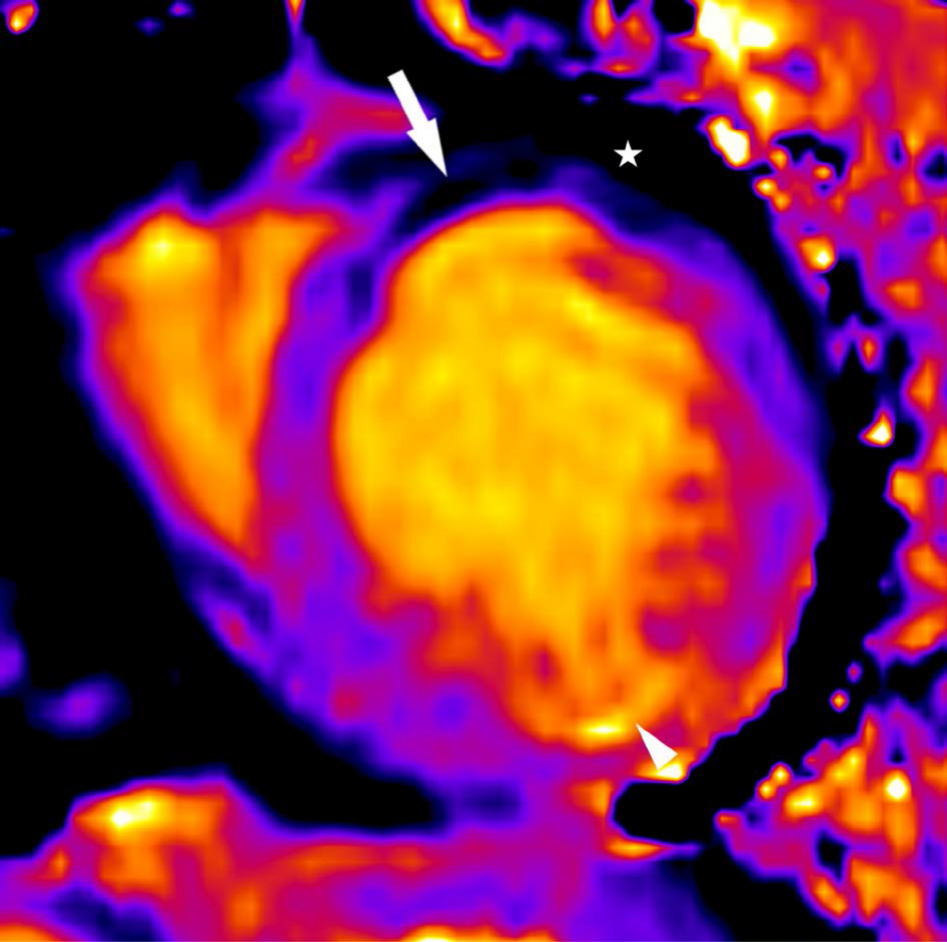
Short axis T1 map shows a midwall area of short T1 (arrow) in the anteroseptal and anterior walls of the left ventricle that is similar to the value of epicardial fat (star). There is an area of prolonged T1 in the inferolateral wall (arrowhead) which corresponds to fibrosis seen on subsequent late gadolinium enhancement imaging.

Tissue characterization including T1 mapping, T2 mapping, and black blood imaging with and without fat suppression confirmed presence of intramyocardial fat. *T*_2_ weighted turbo spin echo black blood imaging without fat saturation (Figure 2, left) identified anteroseptal and anterior segment midwall signal which was effectively nulled by the application of a fat saturation pulse (Figure 2, right). Native myocardial T1 mapping (Figure 3) identified these regions as having very short T1 similar to the epicardial fat. In both the black blood and native myocardial T1 images, the behavior of nearby epicardial fat served as a useful reference against which to compare suspected intramyocardial fat. The regions of suspected intramyocardial fat behaved exactly like the nearby epicardial fat, indicating the similarity of tissue composition.

**Figure 4.**
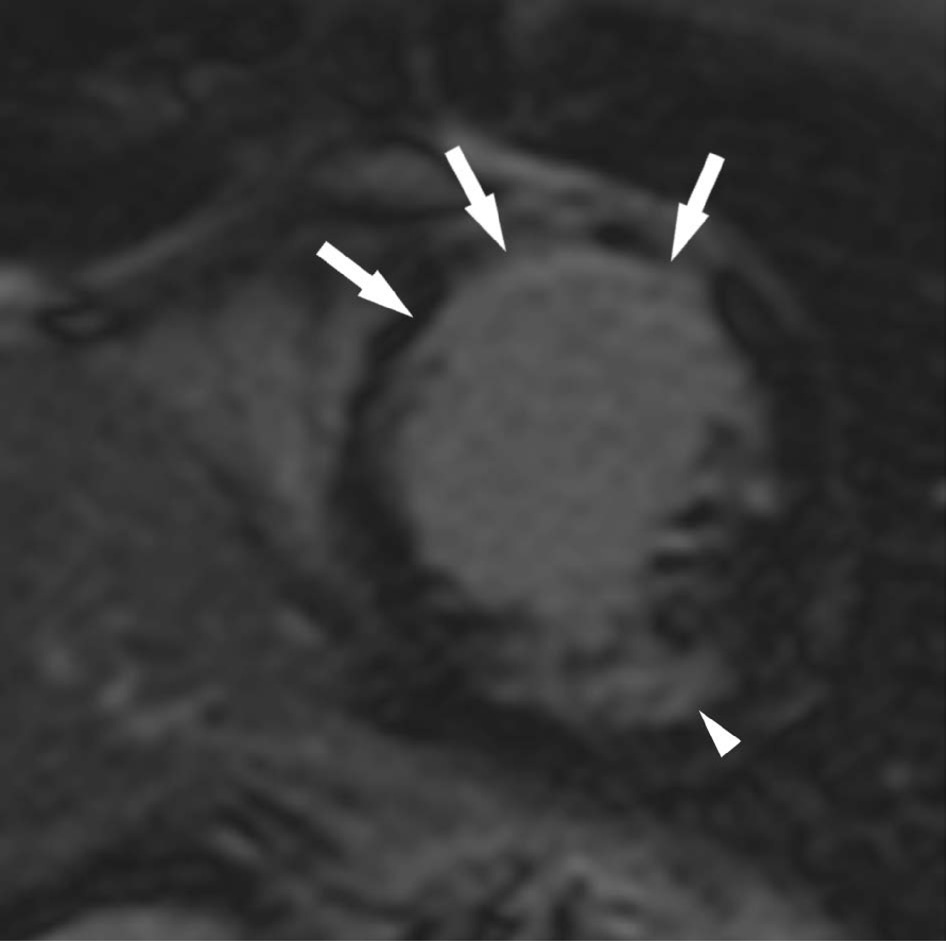
Short-axis magnitude inversion recovery late gadolinium enhancement image of the left ventricle shows areas of subendocardial enhancement within the mid septal and anterior walls (arrows) that is greater in extent than the areas of intramyocardial fat. A separate area of subendocardial fibrosis is present in the inferolateral wall (arrowhead).

Finally, late gadolinium enhancement (LGE) imaging (Figure 4) identified hyperintensity in the anterior and anteroseptal wall. Both fat and fibrosis may appear bright on LGE as both tissues have short T1. However, the hyperintensity on LGE was greater in overall extent than the intramyocardial fat identified on earlier sequences. Thus, the anteroseptal and anterior wall findings on LGE were compatible with the presence of fibrosis in addition to intramyocardial fat. A separate area of fibrosis, not associated with intramyocardial fat, was identified in the inferolateral wall.

## Differential diagnosis

In a patient older than 50 years, differential considerations of intramyocardial fat and fibrosis in the left ventricle in the setting of severe left ventricular dysfunction are broad and include fatty metaplasia of a prior myocardial infarction, arrhythmogenic ventricular cardiomyopathy, dilated cardiomyopathy, and sequela of various diseases such as tuberous sclerosis and Duchenne muscular dystrophy. In this patient, coronary artery disease was excluded. An extensive workup for systemic diseases in the patient was negative, excluding tuberous sclerosis and muscular dystrophy. Normal left ventricular size excludes a dilated cardiomyopathy. The exclusion of these other diseases from the differential diagnosis, T-wave inversions on ECG, presence of intramyocardial fat and fibrosis, as well as severely decreased left ventricular function narrows the differential diagnosis to arrhythmogenic left ventricular cardiomyopathy.

## Treatment and outcome

In summary, this case shows severe left ventricular dysfunction and wall motion abnormalities with areas of intramyocardial fat as well as areas of fibrosis in a patient with an abnormal ECG showing T-wave inversions. There are significant wall motion abnormalities associated with the areas of myocardial fat and fibrosis. An extensive workup excluded other causes of fat deposition within the myocardium. Although genetic testing would have been helpful, it was not performed in our patient. However, the combination of the abnormal ECG as well as the MRI findings was felt to be consistent with arrhythmogenic ventricular cardiomyopathy involving the left ventricle and the patient was treated with a defibrillator.

## Discussion

Arrhythmogenic ventricular cardiomyopathy (AVC) is a rare, inherited myopathy characterized by progressive substitution of the myocardium with fibrofatty tissue. Due to its minimal electrical conductivity, the fatty deposition in the myocardium is associated with arrhythmias.^1–3^ AVC has been associated with mutations in desmosomal genes encoding desmoplakin, plakophilin-2, plakoglobin, desmocollin-2, and desmoglein-2.^2,4^ Van Der Zwaag et al also described the role of a non-desmosomal gene encoding phospholamban, which is also associated with dilated cardiomyopathy, in AVC.^5^ Left ventricular involvement by AVC may be present in advanced arrhythmogenic right ventricular cardiomyopathy (ARVC).^6^ However, left dominant AVC is exceedingly rare and unlike ARVC, it lacks the specific diagnostic criteria, making its diagnosis much more challenging.^2,6,7^

Clinical manifestations of AVC range from asymptomatic to life-threatening ventricular arrhythmia. While uncommon, AVC demonstrates a predilection for athletes and the young. Contrast-enhanced cardiac MR is a powerful imaging tool used to assess patients with early AVC by assessing for regional wall motion abnormalities, cardiac chamber size and function as well as for the presence and distribution of fibrofatty scarring in the myocardium.^2^ Sen-Chowdhry et al reported clinical diagnostic features of arrhythmogenic left ventricular cardiomyopathy (ALVC) based on a study consisting of 42 patients with left dominant arrhythmogenic cardiomyopathy. The septum was involved in more than half of the ALVC cases on MRI, in comparison to advanced-stage ARVC with LV involvement in which septum was usually spared. Fibrofatty scar was distributed in both the LV subepicardium and midmyocardium. The majority of their cohort population had right bundle branch block-type ventricular arrhythmia and inverted T waves in the inferior and lateral leads on ECG.^8^ A high frequency of ventricular ectopic beats and non-sustained ventricular tachycardia (VT) has also been detected on Holter monitoring in other reported cases of left-sided arrhythmogenic ventricular cardiomyopathy.^9,10^

AVC primarily involving the left ventricle may be misdiagnosed as other more common cardiac disorders, such as healed myocardial infarction (MI) and dilated cardiomyopathy.^4,11^ Following an MI, the infarcted myocardium undergoes various structural changes, frequently including fat deposition.^11,12^ Fat infiltration can be seen on imaging studies after at least 6 months after an MI with distribution in the territory of the occluded coronary artery. While CT has demonstrated subendocardial fatty metaplasia in healed MI, subepicardial and midmyocardial fat infiltration have also been seen on MRI of affected patients. This variation might be due to the superior tissue characterization of MRI.^11,13^ Bader et al reported a prognostic role for myocardial fat, which was found to be associated with better survival in patients with and without prior MI.^14^

Idiopathic dilated cardiomyopathy (DCM) is a cause of heart failure in young patients in the absence of coronary artery disease. DCM is identified by systolic dysfunction and dilated chamber size.^13,15^ Fat deposition in DCM is usually located in the midmyocarium.^11,15^ In a study of 124 patients with idiopathic DCM, Lu et al found that the amount of fibrofatty deposition inversely correlates with LVEF.^15^

Other less common conditions that are associated with cardiac fat deposition should also be excluded prior to making the diagnosis of AVC. Cardiac lipoma is a benign neoplasm present as a focal homogeneous encapsulated mass on the imaging studies. This fatty mass is typically located in the subepicardium and subendocardium of the heart.^3,11,13^

Tuberous sclerosis complex (TSC) and Duchenne muscular dystrophy (DMD) are two systemic diseases that can result in myocardial fat deposition. TSC is a genetic multisystem disorder characterized by development of benign tumors frequently seen in the brain, eyes, kidneys, heart and skin. The diagnosis is made based on the presence of definite clinical diagnostic criteria. Fat infiltration in TSC consists of small foci of fat usually located in the left ventricular wall or interventricular septum. The presence of the various systemic findings in these patients helps to specify the diagnosis.^16^ DMD patients usually exhibit cardiac involvement. Fibrofatty infiltration has been shown to typically involve the subepicardial inferolateral wall of the left ventricle. Other manifestations of the disease such as skeletal muscle involvement easily differentiate DMD from others diseases.^11,17^

Symptom reduction, prevention of disease progression and sudden cardiac death, improvement of quality of life by limiting potential arrhythmias and enhancing exercise capacity are critical components of clinical management of AVC patients. Treatment options and recommendations include life style modifications, pharmacologic treatment, catheter ablation in patients with VT, implantable cardioverter defibrillator (ICD) implantation in patients who are at highest risk of sudden cardiac death, and heart transplantation in patients with end-stage heart failure or refractory VT.^18^ These therapeutic options have been recommended for the classic subtype of AVC, however there is lack of a definitive management option that directly applies to predominantly left ventricular involvement by AVC.

## Learning points

- Cardiac MRI is a powerful diagnostic tool that aids in the diagnosis of arrhythmogenic left ventricular cardiomyopathy.
- Predominantly left ventricular involvement by AVC is exceedingly rare and lack of specific diagnostic criteria as well as its potential cardiotoxic effect make its diagnosis challenging and of high importance.
- The differential diagnosis for fat and fibrosis in the left ventricle is broad and includes fatty metaplasia of a prior myocardial infarction, arrhythmogenic ventricular cardiomyopathy, dilated cardiomyopathy, and sequela of various diseases such as tuberous sclerosis and Duchenne muscular dystrophy. These other causes of fat and fibrosis need to be excluded before a diagnosis of arrhythmogenic left ventricular cardiomyopathy can be determined.
